# β-arrestin2 alleviates L-dopa–induced dyskinesia via lower D1R activity in Parkinson’s rats

**DOI:** 10.18632/aging.102574

**Published:** 2019-12-18

**Authors:** Xing-Ru Zhang, Zeng-Rui Zhang, Si-Yan Chen, Wen-Wen Wang, Xin-Shi Wang, Jin-Cai He, Cheng-Long Xie

**Affiliations:** 1 Department of Neurology, The First Affiliated Hospital of Wenzhou Medical University, Wenzhou, China; 2 Department of Neurology, Huzhou Central Hospital, Zhejiang, China; 3 The center of Traditional Chinese Medicine, The Second Affiliated Hospital and Yuying Children's Hospital of Wenzhou Medical University, Wenzhou, China

**Keywords:** β-arrestin2, dopamine receptors, D1R activity, Parkinson's disease, L-dopa–induced dyskinesia

## Abstract

The cause of the L-dopa–induced dyskinesia (LID) has been ascribed to G-protein coupled receptor (GPCR) supersensitivity and uncontrolled downstream signaling. It is now supposed that β-arrestin2 affects GPCR signaling through its ability to scaffold various intracellular molecules. We used the rAAV (recombinant adeno-associated virus) vectors to overexpress and ablation of β-arrestin2. L-dopa-induced changes in expression of signaling molecules and other proteins in the striatum were examined by western blot and immunohistochemically. Our data demonstrated that via AAV-mediated overexpression of β-arrestin2 attenuated LID performance in 6-OHDA-lesioned rodent models. β-arrestin2 suppressed LID behavior without compromising the antiparkinsonian effects of L-dopa. Moreover, we also found that the anti-dyskinetic effect of β-arrestin2 was reversed by SKF38393, a D1R agonist. On the contrary, the rat knockdown study demonstrated that reduced availability of β-arrestin2 deteriorated LID performance, which was counteracted by SCH23390, a D1R antagonist. These data not only demonstrate a central role for β-arrestin2/GPCR signaling in LID, but also show the D1R signal pathway changes occurring in response to dopaminergic denervation and pulsatile administration of L-dopa.

## INTRODUCTION

Parkinson’s disease (PD) is characterized by the serious dopaminergic neurons deficiency in the substantia nigra (SN) of the midbrain, leading to bradykinesia, muscular rigidity and rest tremor et al [1]. The gold standard of drug therapy for PD is oral administration of the dopamine precursor, L-3,4-dihydroxyphenylalanine (L-dopa) [2]. However, after prolonged and pulsatile exposure to L-dopa, PD patients eventually develop a variety of abnormal involuntary movements, termed L-dopa-induced dyskinesia (LID), which represent a major treatment limitation and reduce the quality of life of PD patients [3]. LID is a type of motor complication that occurs in more than 50% of PD patients after 5–10 years L-dopa administration [4]. However, the underlying cellular and molecular key events that result in LID remains unclear right now.

LID is closely correlated with pathological changes in dopaminergic transmissions in the striatum [5]. A large body of evidence showed that increased activity of dopamine D1-receptors (D1R) was imperative for LID development [6]. The increased density of D1R that was found in the striatum of MPTP-treated primates that displayed LID was thought to bring about elevated second-messenger expression and changed in receptor trafficking [7], suggesting that D1R have a specific role in LID expression. Sensitized D1R generated multiple molecular events, such as persistent and intermittent hyper-activation of the cAMP signaling cascade, the induction of immediate early genes and the activation of extracellular signal-regulated kinases (ERK) et al [8, 9]. Darmopil et al demonstrated that the D1R was critical for the development of LID in mice and the underlying molecular changes in the denervated striatum while the D2R had little or no involvement [9]. Shenoy et al displayed that LID was reduced by promoting G-protein coupled receptor (GPCR) desensitization by GPCR kinases (GRKs), followed by binding to arrestins and receptor internalization [10]. Therefore, pharmacological or genetic interventions aimed at reducing abnormal sensitizing D1R maybe a potential alternative approach to control the LID.

The arrestin family (β-arrestin1, β-arrestin2, x-arrestin, and s-arrestin), are cytosolic proteins that increase GPCR internalization and trafficking in GRK-dependent manners [11]. Among them, β-arrestin2 is one of two arrestin isoforms in the brain can transduce GPCR signals by forming protein complexes with signaling molecules downstream of G protein to mediate GPCR desensitization or recycling et al [12]. One previous research showed that co-expression of β-arrestin2 and GRK3 was essential for rapid desensitization of CB1 receptor-mediated potassium currents following exposure to WIN55,212-2 in Xenopus Oocytes [13]. Similarly, inactivation β-arrestin2 attenuated desensitization of WIN55,212-2-mediated inhibition of glutamatergic neurotransmission in hippocampal neurons [14]. Although β-arrestin2 can transduce multiple signals in cells, little is known about their participation in PD or LID. Only one study reported that β-arrestin2 overexpression reduced LID performance while maintaining the therapeutic effect of L-dopa in knock-out PD mice [15]. In the present study, therefore, we aim to investigate the effect of genetic overexpression/deletion of β-arrestin2 in the striatum influence the occurrence of abnormal involuntary movements (AIM) score and the motor function. Meanwhile, we also further inspect the effect of β-arrestin2 on LID via lower D1R activity using selective D1R agonist/antagonist.

## RESULTS

### AAV-Mediated β-arrestin2 overexpression alters AIMs induced by L-dopa in hemi-parkinsonian rats

**Figure 1:**
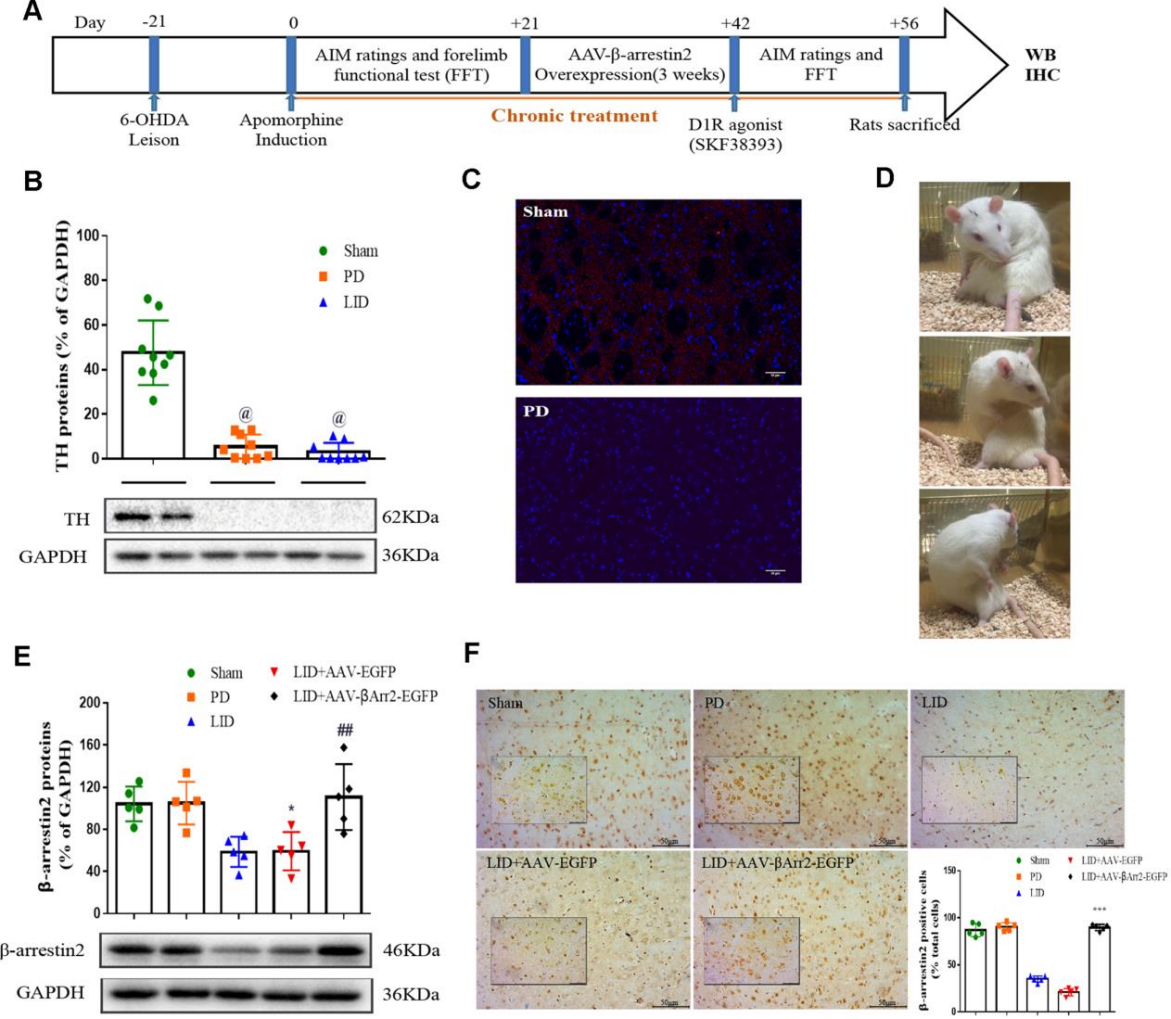
**Recombinant adeno-associated virus (AAV)-induced β-arrestin2 overexpression (β-arrestin2^+/+^) in the unilateral 6-OHDA-lesioned striatum of SD rats.** (**A**) Schedule of experiment in β-arrestin2^+/+^ rats. At the beginning of the studies, the rats (n=56) was injected unilaterally with 6-OHDA in the right medial forebrain bundle (MFB). After three weeks, lesion efficacy was assessed by the rotations after the use of apomorphine for the following induction of L-dopa-induced dyskinesia (LID). The β-arrestin2 overexpression rat models on the base of LID were constructed immediately and followed by intraperitoneal injection of D1R agonist (SKF38393) once a day. All groups of rats except PD and sham were injected with L-dopa (15 mg/kg, i.p.) plus benserazide (3.75 mg/kg, i.p.). Additionally, rats in the PD group and sham group were treated physiological saline. After the last drug administration, all rats were sacrificed in two hours and fetched the lesioned corpus striatum for the following WB, IHC and IFC. (**B**) Tyrosine hydroxylase (TH) protein levels was markedly decreased by nearly 90% in the PD or LID rats compared with sham group by western blot (n=4 for each group). (**C**) Fluorescence slice of coronal plane from striatum of rats between sham and PD groups stained with TH (n=4 for each group). Scale bar represents 100 um. (**D**) Still images showing L-dopa-induced dyskinesia in a parkinsonian rat. (**E**) AAV-β-arrestin2 expression (β-arrestin2^+/+^) relative to GAPDH level in five groups by WB in the lesioned striatum (n=4 for each group). The experiments for Figure 1B and Figure 1E were performed together with the same Sham, PD and LID tissues for western blot; (**F**) AAV-β-arrestin2 expression (β-arrestin2^+/+^) in five groups by IHC in the lesioned striatum (n=4 for each group). Scale bar represents 100 um. The experiments for Figure 1C and Figure 1F were performed together with the same Sham and PD for immunohistochemistry. @ P < 0.0001 vs sham group; * p < 0.05, ## P > 0.05 vs sham group; ***p<0.001 vs LID+EGFP group (n = 4 for each group, ANOVA followed by LSD post-hoc comparisons).

**Figure 2:**
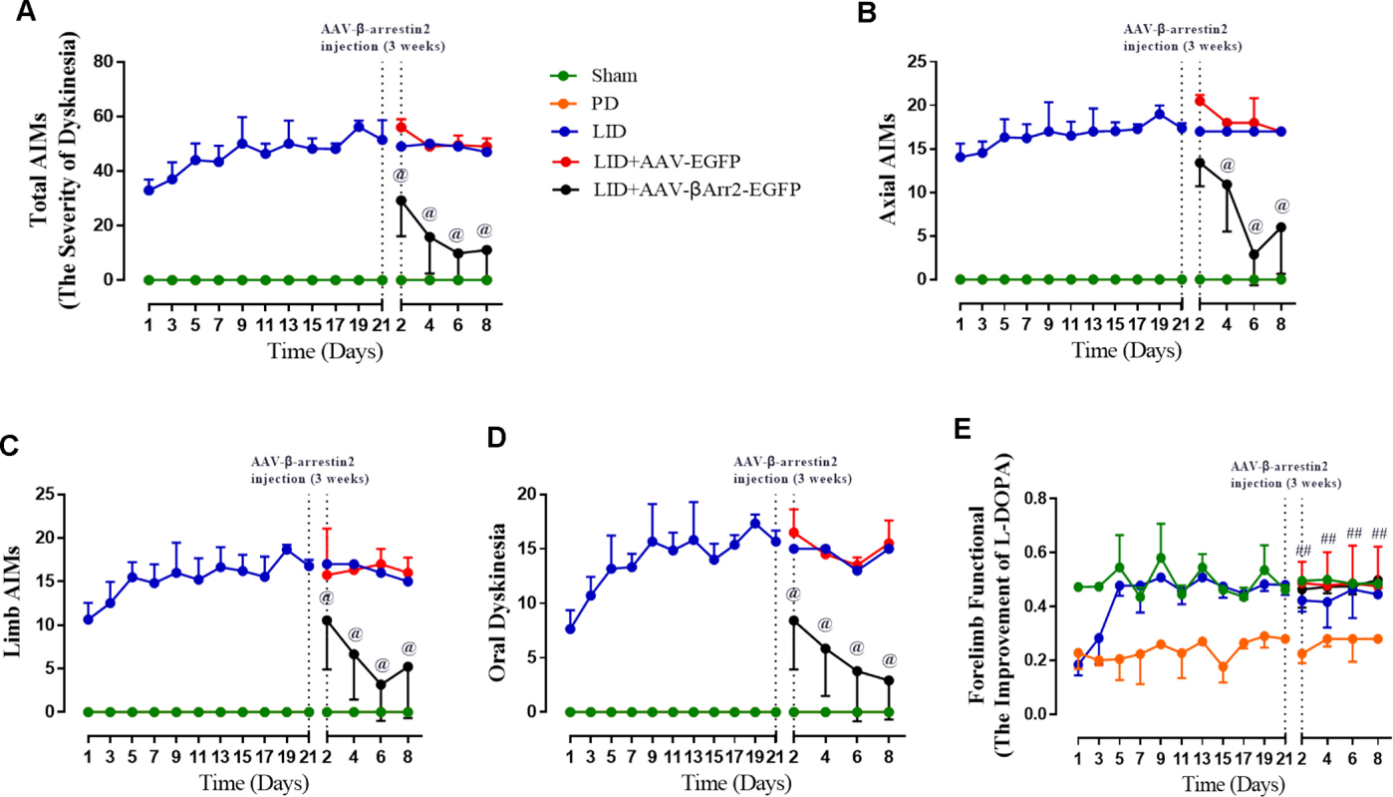
**AAV-Mediated β-arrestin2 overexpression reduces AIMs induced by L-dopa in 6-OHDA-lesioned rats.** Rats were rated for AIMs on days 1, 3, 5, 7, 11, 13, 15, 17, 19 and 21 and carried on testing on days 2, 4, 6 and 8 after the striatum-targeted injection of β-arrestin2^+/+^ (n=8 for each group, total n=40 rats). (**A**) Total AIM scores measured after L-dopa or saline injection in all groups; (**B**) axial AIM score; (**C**) limb AIM score; (**D**) orolingual AIM score; (**E**) Forelimb functional test. Data are displayed as average ± standard error; @ P<0.0001 vs LID, ## P > 0.05 (n = 8 for each group, Kruskal Wallis followed by Dunn’s test for multiple comparisons or two-tailed unpaired t test).

To build the generation of abnormal movements in PD by repeated L-dopa administration, we utilized a well settled hemi-parkinsonian rat model in which rats were first rendered hemi-parkinsonian by unilateral injection of 6-OHDA into the MFB. Figure 1A showed the experimental design of first section (n=56). The extent of 6-OHDA-induced dopamine neurons alterations in striatum was evaluated 21 days after surgery by western blotting and immunofluorescence techniques with an antibody raised against TH. We found the TH levels were decreased by more than 90% in the lesioned hemisphere of 6-OHDA-lesioned rats treated or not with L-dopa when compared with the sham group (n=4 for each group, P<0.0001, Figure 1B and 1C) and meanwhile there was no difference in the non-lesioned between three groups (Supplementary Figure 1). The severity of dopamine depletion was evaluated by measuring the contralateral rotation response to apomorphine (0.5 mg/kg, i.p., Figure 1D). There was a striking reduction in β-arrestin2 proteins and immunostaining in the dopamine-depleted striatum of rats treated with L-dopa. We constructed AAV encoding EGFP (control) or rat β-arrestin2 tagged with EGFP for easy detection and found AAV-β-arrestin2-EGFP can completely changeover the β-arrestin2 proteins and immunostaining by L-dopa administration (n=4, P > 0.05 vs sham or PD groups, Figure 1E and 1F). We tested whether overexpression of β-arrestin2 would influence preexisting dyskinesia. While in AAV-β-arrestin2-EGFP injected rats dyskinesia scores (Total AIMs) dropped significantly from week 3 until week 4 postsurgery, when compared with LID and LID + AAV-EGFP groups (n=8 for each group, two-tailed unpaired t test: p < 0.0001, Figure 2A). The results showed that PD rats treated with pulsatile L-dopa developed significant axial (Figure 2B), limb (Figure 2C) and orolingual AIMs (Figure 2D) compared with sham or PD rats receiving saline. Moreover, it is noteworthy that animals receiving intrastriatal AAV-β-arrestin2-EGFP infusion in combination with L-dopa treatment exhibited significantly lower axial (p < 0.0001, Figure 2B), limb (p < 0.0001, Figure 2C) and orolingual AIMs (p < 0.0001, Figure 2D) compared with LID+EGFP group. We also confirmed that co-injected AAV-β-arrestin2-EGFP and L-dopa, did not alter the beneficial motor movement effect of L-dopa treatment by the means of FFT test (p > 0.05, Figure 2E).

### The anti-dyskinetic effect of β-arrestin2 overexpression is overcome by D1R agonist

**Figure 3:**
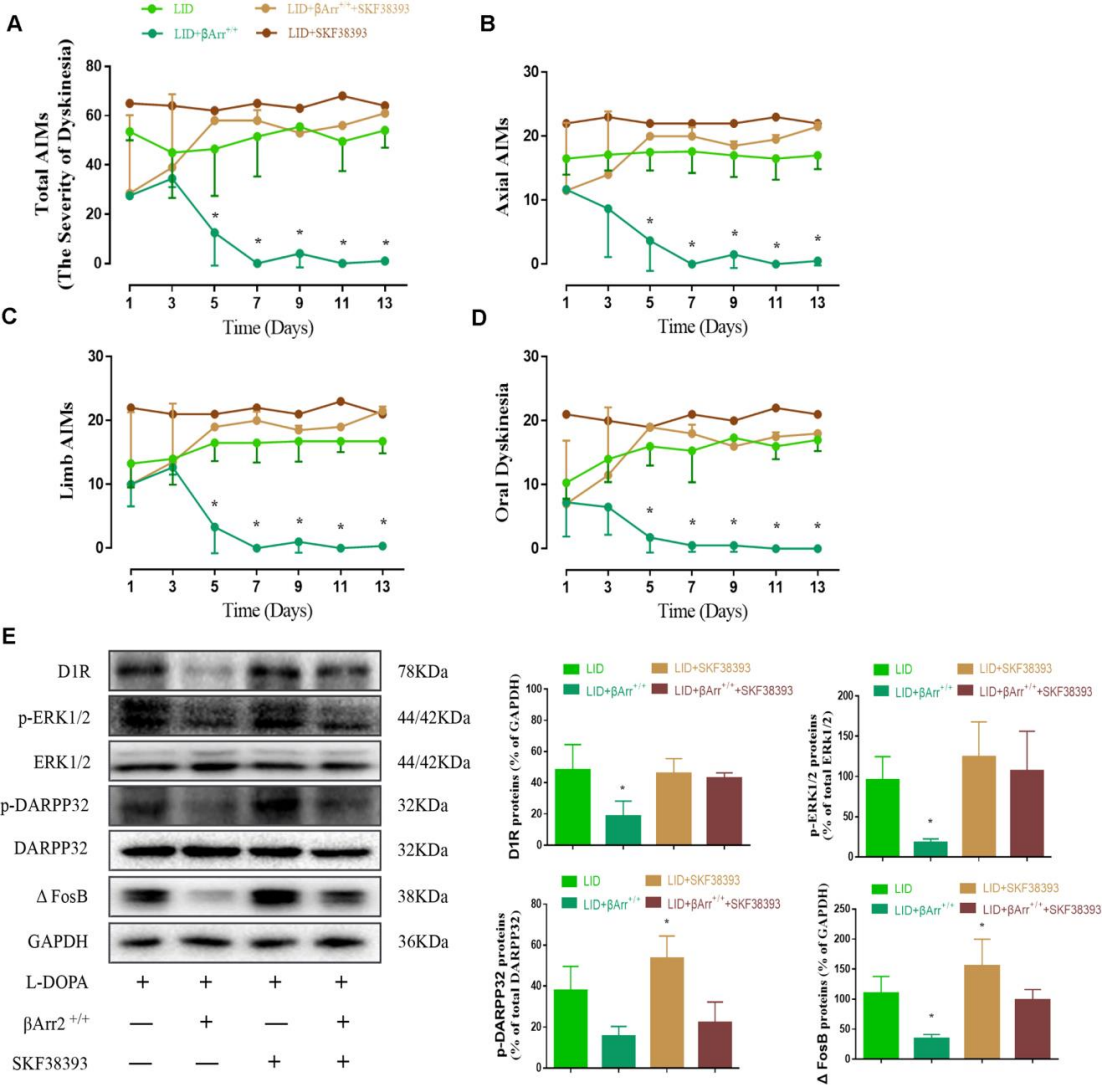
**AAV β-arrestin2 overexpression and LID groups respectively followed by intraperitoneal injection of D1R agonist (SKF38393) or saline.** Rats were rated for AIMs on days 1, 3, 5, 7, 9, 11 and 13 after the injection of SKF38393. Collecting the striatums of the following four groups to prepare tissue homogenate for WB: LID, LID plus β-arrestin2^-/-^, LID plus SCH23390, and LID plus β-arrestin2^-/-^ plus SCH23390. (**A**) Total AIM scores; (**B**) axial AIM score; (**C**) limb limb; (**D**) orolingual AIM score (n = 4 for each group, total 4*4=16); (**E**) The protein level of D1R, phosphor-ERK1/2, phosphor-DARPP32 and FosB by WB. Data are displayed as mean ± standard error of mean; * P<0.05 vs LID + βArr^+/+^ + SKF38393 group (n = 4 for each group, Kruskal Wallis followed by Dunn’s test for multiple comparisons in AIM scores, ANOVA followed by LSD post-hoc comparisons in proteins detection).

Repeated pulsatile L-dopa injection to 6-OHDA-lesioned rats leads to a progressive increase in the total AIMs. The total AIM score was markedly reduced in animals expressing β-arrestin2 (n = 4 for each group, total 4*4=16, Figure 3A). Thus, the increased availability of β-arrestin2 alleviates already established dyskinesia. And then we found that the effect of β-arrestin2 overexpression was obviously reversed by SKF38393 (1.5 mg/kg), a D1R agonist, in terms of total AIMs (p < 0.05 vs LID and LID + β-arrestin2^+/+^; Figure 3A). Analogously, we found this seemed to be the same tendency in axial AIM (p < 0.05, Figure 3B), limb AIM (p < 0. 05, Figure 3C) as well as orolingual AIM (p < 0. 05, Figure 3D). Meanwhile, β-arrestin2 overexpression- associated decreases in D1R level, FosB expression, DARPP-32 and ERK1/2 phosphorylation were also prevented by SKF38393 in the striatum of dyskinetic animals (n = 4 for each group, p < 0.05 vs LID and LID+ β-arrestin2^+/+^; Figure 3E). We have also investigated the D2R expression and the level of DR2 was decreased in LID rats but not changed by AAV-β-arrestin2 or AAV-EGF (Supplementary Figure 2).

### AAV-mediated β-arrestin2 ablation deteriorates AIMs induced by L-dopa

**Figure 4:**
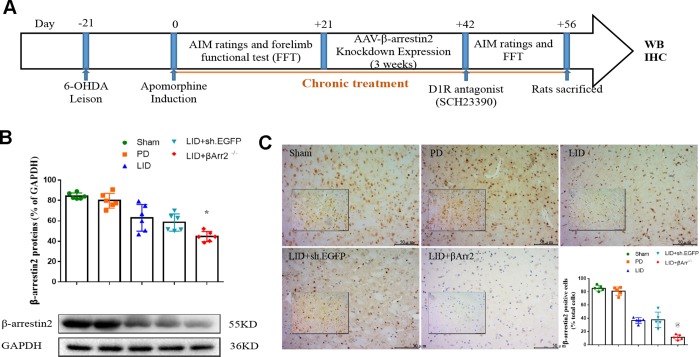
**Recombinant AAV-mediated β-arrestin2 knock-down (β-arrestin2^-/-^) in 6-OHDA-lesioned rats treated with L-dopa.** (**A**) The whole process of the second part experimental design (n=56); (**B**) AAV-β-arrestin2 knock-down (β-arrestin2^-/-^) relative to GAPDH level in five groups by WB in the lesioned striatum (n = 4 for each group, total n=4*5=20); (**C**) AAV-β-arrestin2 knock-down (β-arrestin2^-/-^) in five groups by IHC in the lesioned striatum. Scale bar represents 100 um. @ P<0.0001, * p<0.05 vs LID + sh. EGFP group (n = 4 for each group, total 4*5=20, ANOVA followed by LSD post-hoc comparisons).

**Figure 5:**
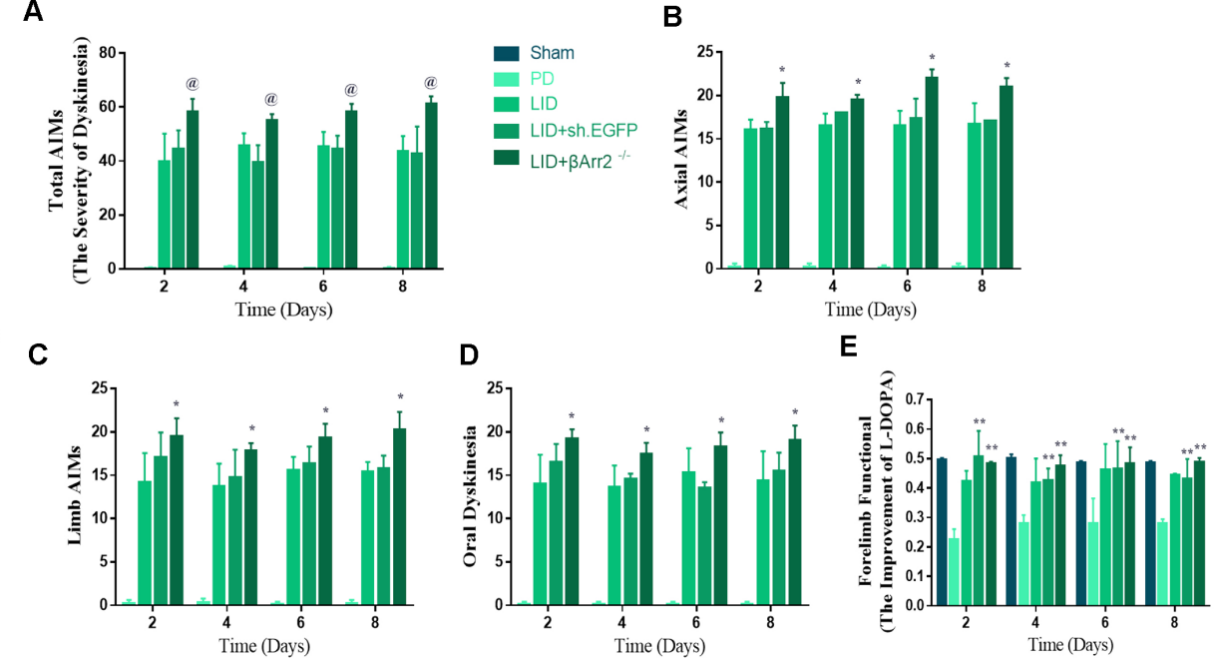
**AAV-Mediated β-arrestin2 knock-down (β-arrestin2^-/-^) aggravates AIMs induced by L-dopa in 6-OHDA-lesioned rats.** Rats were rated for AIMs on days 2, 4, 6 and 8 after the striatum-targeted injection of β-arrestin2^-/-^ (total n=40 rats). (**A**) Total AIM scores measured after L-dopa or saline injection in all groups; (**B**) axial AIM score; (**C**) limb AIM score; (**D**) orolingual AIM score; (**E**) Forelimb functional test. @ P<0.0001, * p < 0.05 vs LID + sh.EGFP group; ** P<0.01 vs PD group (n = 8 for each group, Kruskal Wallis followed by Dunn’s test for multiple comparisons).

To assess the role of endogenous β-arrestin2, we tested whether knockdown of β-arrestin2 with AAV-delivered shRNA would influence the behavioral effects of L-dopa and a luciferase shRNA as a negative control. Figure 4A showed the experimental design of second section. 6-OHDA-lesioned rats were treated with L-dopa for 14 days after virus injection (incubation three weeks). Similarly, there was a striking decline in the field of β-arrestin2 proteins and immunostaining in the striatum of LID rats treated compared with PD and sham groups (p < 0.05, Figure 4B and 4C). Namely, the β-arrestin2 concentration was distinctly decreased by the β-arrestin2 shRNA as compared to the control adenovirus. And we found AAV-sh. β-arrestin2 (LID + β-arrestin2^-/-^), rather than LID + sh. EGFP, can further decrease the β-arrestin2 proteins (n = 4 for each group, p < 0.05, Figure 4B) and immunopositive neurons (n = 4 for each group, p < 0.0001, Figure 4C) compared with LID group. Next, we tested whether ablation of β-arrestin2 in the lesioned striatum influence the dyskinesia performance and found β-arrestin2 knockdown exacerbated the progressive increase in total AIMs (n = 8 for each group, p < 0.0001 vs LID and LID + sh. EGFP, Figure 5A). Analogously, we found this seemed to be the same trend in axial AIM (p < 0.05 vs LID and LID + sh. EGFP, Figure 5B), limb AIM (p < 0.05 vs LID and LID + sh. EGFP, Figure 5C) as well as orolingual AIM (p < 0.05 vs LID and LID + sh. EGFP, Figure 5D) by AAV-sh. β-arrestin2 injection. Meanwhile, β-arrestin2 knockdown did not change the beneficial motor movement effect of L-dopa treatment in terms of FFT test (Figure 5E).

### The effect of β-arrestin2 ablation is counteract by D1R antagonist

**Figure 6:**
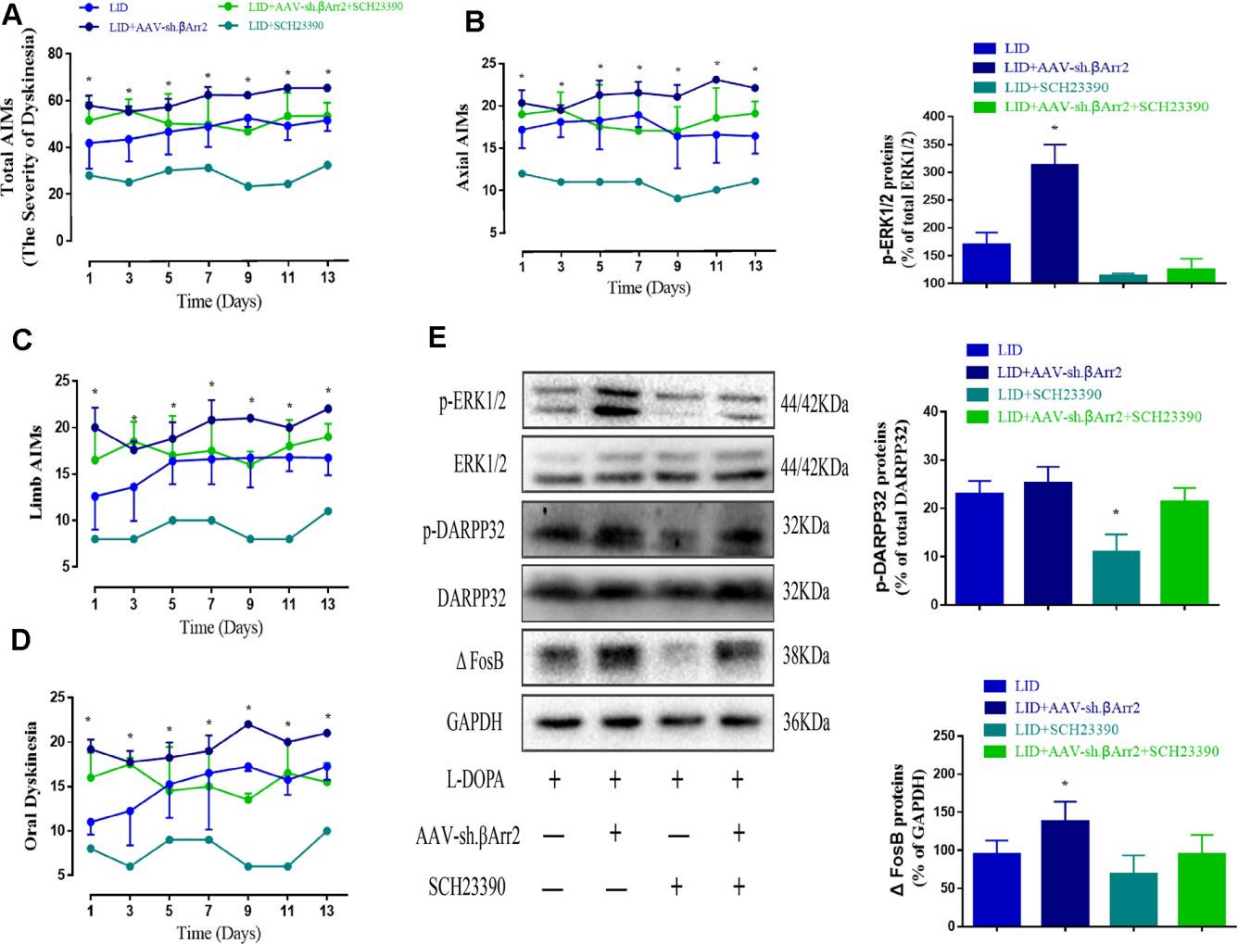
**AAV β-arrestin2 knock-down (β-arrestin2^-/-^) and LID groups respectively followed by intraperitoneal injection of D1R antagonist (SCH23390) or saline.** Rats were rated for AIMs on days 1, 3, 5, 7, 9, 11 and 13 after the injection of SCH23390 and L-dopa. Samples from the striatum of LID, LID plus β-arrestin2^-/-^, LID plus SCH23390, and LID plus β-arrestin2^-/-^ plus SCH23390 were collected and different downstream markers of the D1R signal pathway were measured by WB. (**A**) Total AIM scores; (**B**) axial AIM score; (**C**) limb AIM score; (**D**) orolingual AIM score (n = 4 for each group, total 4*4=16); (**E**) The protein level of phosphor-ERK1/2, phosphor-DARPP32 and FosB by WB. Data are displayed as mean ± standard error; * P < 0.05 vs LID + βArr^-/-^ + SCH23390 group (n = 4 for each group, Kruskal Wallis followed by Dunn’s test for multiple comparisons in AIM scores, ANOVA followed by LSD post-hoc comparisons in proteins detection).

Figure 6 showed β-arrestin2 knockdown augment the dyskinesia behavior was apparently reversed by SCH23390 (0.25 mg/kg), a D1R antagonist, in terms of total AIMs (n = 4 for each group, total 4*4=16, p < 0. 05 vs LID + β-arrestin2^-/-^, Figure 6A), axial AIM (p < 0.05 vs LID + β-arrestin2^-/-^, Figure 6B), limb AIM (p < 0. 05 vs LID + β-arrestin2^-/-^, Figure 6C) and orolingual AIM (p < 0. 05 vs LID + β-arrestin2^-/-^, Figure 6D). Postmortem examination of the infected striatum revealed that β-arrestin2 knockdown was significantly increased the level of D1R, FosB expression, DARPP-32 and ERK1/2 phosphorylation compared with LID group (Figure 6E). As expected, all these signal pathway molecular markers were changeover by SCH23390 (n = 4 for each group, Figure 6E), indicating β-arrestin2 impacted LID performance maybe via D1R signal.

## DISCUSSION

The primary of PD motor symptom treatment since the 1960s is the DA precursor L-dopa and will likely still so for a while until better compounds are developed [15]. Our data demonstrated that via AAV-mediated overexpression of β-arrestin2 attenuated LID performance in rodent models. β-arrestin2 suppressed LID in dyskinetic rats without compromising the antiparkinsonian effects of L-dopa. Moreover, we also found that the anti-dyskinetic effect of β-arrestin2 was reversed by SKF38393, a D1R agonist. On the contrary, the rat knockdown study demonstrated that reduced availability of β-arrestin2 promoted LID performance in term of AIM scores, which was counteracted by SCH23390, a D1R antagonist. Collectively, these data displayed increased level of β-arrestin2 via decrease D1R activity helps to control LID without sacrificing the antiparkinsonian benefits of L-dopa.

Lack of dopamine in PD results in multiple changes in the dopamine mediated signaling [16]. The premier dysregulation of signaling pathways is further exaggerated by chronic/pulsatile L-dopa treatment, eventually leading to dyskinesia [17]. The cause of the dyskinesia, at least in part, has been ascribed to GPCR supersensitivity and uncontrolled downstream signaling and neuronal activity [18, 19]. Hence, the challenge is to cut down the supersensitivity and signaling that relieves LID while retaining the anti-parkinsonian activity of the drug, which is also mediated by dopamine receptors would be ideal. It is now supposed that β-arrestin2 adjust GPCR signaling through their ability to scaffold various intracellular molecules such as kinases and phosphatases [20]. Nikhil and colleague reported that either a genetic or pharmacological intervention to enhance β-arrestin2, which can limit G protein-dependent D1R signaling and reversed the LID performance [17, 21], in consistent with our finding in this paper. Here, we showed that the level of β-arrestin2 was decreased in the lesioned hemisphere, indicating a link between lower β-arrestin2 availability and dyskinesia. It is reasonable that low level of β-arrestin2 weaken the GPCR internalization in LID rats and overexpression β-arrestin2 promoting desensitization of GPCR and offset the LID performance. Meanwhile, AAV-shRNA mediated β-arrestin2 knockdown in the striatum further exacerbated the decrease in the β-arrestin2 expression and deteriorated the behavioral consequences (AIM score) of dopamine depletion and L-dopa treatment, supporting the role of low β-arrestin2 in LID. However, one limitation should be highlight is when a knocking down approach is performed, especially in “in vivo” models, checking AAV diffusion as well as the expression levels of other proteins is pivotal (e.g. β-arrestin1, ERK1/2 et al). Conversely, AAV-mediated β-arrestin2 overexpression, doubled β-arrestin2 concentration, significantly improved dyskinetic behavior, which in good agreement with the work by Nikhil et al. The results present in the paper underscore an important functional role of β-arrestin2 in signaling mechanisms underlying LID. Indeed, these data not only confirm the use of the 6-OHDA-lesioned model and ALO AIM recording as a correlate for LID but also establish β-arrestin2-dependent functions as an effective target for LID in L-dopa therapy in PD. Moreover, we found either β-arrestin2 overexpression or knockdown both did not change the beneficial motor movement effect of L-dopa treatment in terms of FFT test, which were inconformity with previous results. Therefore, in future, we should utilize more behavior tests to assess Parkinsonism performance.

Dopamine receptors (DAR) belong to the superfamily of GPCR and, similar to many other members of the GPCR family [22]. Ligand-activated DAR are phosphorylated by one or more GRKs, and then bound by multipurpose scaffolding arrestin proteins that definitely recognize phosphorylated GPCRs [23]. These arrestin proteins further arrest G proteins by competing for overlapping binding sites on the receptor [24]. Significantly, when one arrestin protein binds to a receptor, it also starts GPCR internalization and other events ultimately generating the receptor recycling or downregulation [25]. In our paper, β-arrestin2 likely changes L-dopa-dependent behavior by promoting desensitization of G protein-dependent dopamine receptors. Although both dopamine receptor subtypes (D1R and D2R) are involved in LID [9], the D1R seems to play a particularly important role [26]. In hemiparkinsonian rats, we found that β-arrestin2 promoted D1R internalization and suppressed the L-dopa-induced up-regulation of FosB expression, DARPP-32 and ERK1/2 phosphorylation, which attributed to the enhanced D1R signaling. Conversely, β-arrestin2 knockdown increased the level of FosB expression, DARPP-32 and ERK1/2 phosphorylation in LID rats. Thus, the mode of behavioral and molecular effects is keeping with the conclusion that β-arrestin2 relieved LID by accelerating the desensitization of dopamine receptor subtype (D1R) and normalizing biochemical markers. However, to date, there is still no well recognized LID model *in vitro* and primary striatal neurons can be a tool to reflect LID environment in a certain extent. Unfortunately, the levels of D1R signal pathway markers were not directly tested in this paper in primary striatal neurons, which can be investigated in a future study.

These data not only demonstrate a central role for β-arrestin2/GPCR signaling in LID, but also disclose the D1R signal pathway changes occurring in response to dopaminergic denervation and pulsatile administration of L-dopa. These findings further reveal that targeting LID can be achieved without reducing the antiparkinsonian properties of L-dopa when specifically promoting β-arrestin2 expression.

## MATERIALS AND METHODS

### Animals

All studies were approved through the Ethical Committee of Wenzhou Medical University (Wenzhou, China). Adult male Sprague-Dawley (SD) rats weighting 200–250g that were 9–11 weeks old at the start of the experiment were used in all experiments (N=112) and the detailed number of rats used in each experimental group can be found in Figure legends. All rats were housed under controlled lighting conditions in a 12/12 hours’ light/dark cycle and comfortable temperature of 22–26°C with water and food available *ad libitum*. Animals had at least one week for acclimatization before the stereotaxic procedure. All animals were used in compliance with the Institutional Review Board of Wenzhou Medical University and were performed based on the guideline of the National Institutes of Health for the care and use of laboratory animals (NIH publication No.80-23). All efforts were made to reduce animal numbers used to the minimum required for valid statistical analysis.

### Virus construction and preparation

Overexpression or silence of β-arrestin2 coupling with EGFP was based on constructing the recombinant adeno-associated virus (AAV) expression vectors. The whole production and purification procedures are described in detail in previous papers (Levodopa/Benserazide PLGA Microsphere Prevents L-Dopa– Induced Dyskinesia via Lower β-Arrestin2 in 6-Hydroxydopamine Parkinson’s Rats) [21]. Viruses containing overexpressed/silence β-arrestin2 vectors groups (β-arrestin2^+/+^, β-arrestin2^-/-^, respectively) and AAV empty vectors groups were infused stereotactically into the unilaterally striatum of lesioned side at a rate of 0.1 ul/min for 10 min (final volume 1.0 ul/site) and the micro-syringe was held in place for an additional 10 min before being slowly withdrawn. The constructed plasmid and complete plasmid were diverted into AAV respectively and the final virus titer was 1.37E+13 v.g./ml, and the titer used in the study had been diluted to 1.37E + 12 v.g./ml.

### Induction of L-dopa-induced dyskinesia (LID)

Rats were anesthetized with 1% pentobarbital sodium (40 mg/kg, i.p.) and were installed on a stereotaxic apparatus in order to target the medial forebrain bundle (MFB) relative to Bregma according to the Paxinos and Watson rat brain atlas [27]: 1) AP, -4.4 mm, ML, -1.2 mm, DV, -7.8 mm; 2) AP, -3.7 mm, ML, -1.7, DV, -7.8. The tooth bar was set to -2.4 mm. 6-OHDA (32 ug in 4 ul) in 0.2% ascorbic acid was infused unilaterally at 1 ul/min into the MFB. Body temperature was maintained at 37 ^o^C using a heating pad. Three weeks after surgery until the first appearance of Parkinsonism. PD models were selected by the rotations after the use of apomorphine (0.5 mg/kg, i.p.) and the rats displaying more than 7 full body turns/min toward the opposite side of the lesioned side were included. After stabilisation of the parkinsonian phenotype, dyskinesia, both choreiform and dystonic. Rats were administrated twice-daily (9:00 and 15:00) with L-dopa (15 mg/kg, i.p.) plus benserazide (3.75 mg/kg, i.p.) for 3 weeks to induce LID.

### Drug treatment

After LID established, a total volume of 2 ul of virus was injected into each animal and then incubated three weeks again (1 ul per side at two different rostrocaudal sites). LID rats were used to assess whether AAV-mediated overexpression or ablation of β-arrestin2 in motor striatum modulates existing LID performances (ALO AIM score). Intracerebral injections of AAV into the ipsilateral striatum relative to Bregma as follows: 1) anterior-posterior (AP), +0.9 mm, medial-lateral (ML), -4.5 mm, dorsal-ventral (DV), -5.0 mm relative to Bregma; 2) AP, +0.5 mm, ML, -2.5 mm, DV, -4.2 mm according to the rat brain atlas. To maintenance of transgene expression 3 weeks after virus infusions, behavioral experiments were carried out in different groups. SKF38393 or SCH23390 (D1R agonist, D1R antagonist, respectively) was dissolved in saline and administered 30 min prior to L-dopa/Benserazide intake for 2 weeks. The dose of SKF38393 (1.5 mg/kg) or SCH23390 (0.25 mg/kg) used were based by previous papers [28, 29].

### AIM ratings

L-dopa induced abnormal involuntary movements (ALO AIMs) were assessed by two lab members blind to treatment group as described elsewhere [30]. The following 3 subtypes of ALO AIMs were appraised [31]: axial AIMs: twisting movements or dystonic posturing of the head, neck or trunk towards the side contralateral to the lesion; limb AIMs: circular or jerky movements of the forelimb; and orolingual AIMs: empty chewing movements, jaw opening and tongue protrusion, accompanied by twitching of the facial musculature. For each rat, the maximum theoretical score per monitoring session was 4*3*6=72 regarded as ALO AIM scores.

### Forelimb functional test (FFT)

A quantitative assessment of locomotor activity using FFT was performed during L-dopa treatment, which was carried out 90 min after L-dopa administrated and was used as an index of parkinsonian disability score. The test was performed as our previous study [32]. All test processes were carried out in a glass beaker with 35 * 25 cm to count bilateral forelimb use during vertical stand exploration. During 60 min observation, all weight-bearing wall contacts of the bilateral forepaws were counted every 20 min. The final percentage was expressed as using of contralateral forelimb compared with the total number of bilateral limb contacts [33].

### Western blot and immunohistochemistry

The procedure of the western blot, IHC or IFC were according to our previous paper [21]. After the last drug administration (two hours later), all rats were decapitated under 1% pentobarbital sodium, and their brains were collected immediately and two-tailed corpus striatums exhibited radial pattern were cut out in EP pipes on dry ice. Lesioned striatal tissues were homogenated in protein extraction mixed solution comprised of RIPA Lysis Buffer, and freshly-added protease inhibitor cocktail (solarbio) and 100mM PMSF (Beyotime Institute of Biotechnology). The prepared supernatant was placed in new pipes after centrifugation at 12,000 × g for 5 min at 4°C and total and membrane-enriched proteins were contained. Then, protein quant was determined by BCA protein determination method. Experimented corpus striatum samples of every rats from each treatment group were loaded on 8–12% sodium dodecyl polyacrylamide gels 35 ug of protein. Briefly, SDS-PAGE electrophoresis was used to separate the protein and transferred to Polyvinylidene Fluoride (PVDF, pore size: 0.45um) membranes. The membrane was blocked with 5% milk in Tris-buffered saline–Tween 20, and incubated with primary antibodies respectively overnight at 4°C, polyclonal rabbit anti-Tyrosine Hydroxylase antibody (1:1000; Millipore, *No.ab152*), polyclonal rabbit Anti-Beta Arrestin 2 (1:1000; Abcam, *No.ab31294*), polyclonal rabbit anti-DR1 IgG (diluted 1:1000; Millipore), monoclonal rabbit anti-ERK1/2 [1:1000; Cell Signaling Technology (CST)], monoclonal rabbit anti- phosphor-ERK (pT202/pY204, 1:1000; CST), monoclonal rabbit anti-DARPP32 (1:1000; CST), polyclonal rabbit anti-phosphor-DARPP32 (Thr34) (1:1000; Sigma-Aldrich), monoclonal rabbit anti-FosB (1:1000; CST) and polyclonal rabbit anti-GAPDH antibody (1:5000; Bioworld Technology). Then, the membrane was incubated with anti-rabbit horseradish peroxidase IgG (1:1000; Beyotime Institute of Biotechnology) for 1 h at room temperature after 3 times washing using Tris-buffered saline–Tween 20 and immunoreactive bands quantified was based on secondary binding chemiluminescence detection system via Quantity One software (Image Lab, Bio-Rad). Rats were sacrificed immediately after the last L-dopa injection and the behavioral effect via decapitation and perfused transcardially with 0.9% NaCl followed by cold 4% formaldehyde. Their brains kept intact were prepared in 4% formaldehyde (4°C), then soaked in 10%, 20%, 30% sucrose in turn and sink right to the bottom. Brains were frozen and stored at −80 °C until frozen section. 5-8 μm-thick microtome-cryostat cut coronal rat brain sections were collected and processed for IHC and IFC. For IHC experiments, brain sections were then added in 3% hydrogen peroxide to enclose endogenous peroxidase. For antigen retrieval, the sections were rinsed (3*5 min) in 0.1M PBS and transferred to 10 mM sodium citrate solution while gently stirring at 80 °C for 30 min. Slices were incubated overnight at 4 °C temperature with the primary antibody: rabbit Beta Arrestin2 (1:200; Abcam, *No.ab31294*). After rewarming for 45 min at 37 °C and washing in PBS for 3 times, brain sections were incubated for 1 hour in secondary goat anti-rabbit antibody (diluted 1:200; Beyotime Institute of Biotechnology) and then colorated in DAB mixed solution. Image acquisition was processed under microscope and the tissue signal has been quantified using Image-Pro Plus 6.0. In terms of IFC, striatum sections were incubated overnight at 4 °C in the primary antibody solution, rabbit anti-Tyrosine Hydroxylase (1:200; Millipore, *No. ab152*) for fluorescent double-labeling method after 5% BCA in PBS. Then, followed scrubbing by PBS for 3 times slices were hatched away from light in FITC-conjugated goat anti-rabbit IgG (1:1000, Beyotime Institute of Biotechnology) at room temperature for 1 hour. Whereafter, sections were mounted by anti-Fluorescent quenching solution and observed under fluorescence microscope.

### Statistical analysis

Data were expressed as the mean ± standard error of mean (SEM). The behavioral measurements for AIM and FFT are non-parametric and were analyzed using a Kruskal Wallis followed by Dunn’s test for multiple comparisons in the case of comparing data over multiple days. Neurochemical data conformed to normal distribution were analyzed by one-way analysis of variance (ANOVA) followed by LSD post-hoc comparisons when appropriate. The value of P < 0.05 was considered significant.

## Supplementary Material

Supplementary Figures
