# Supplementary material for: β-arrestin2 alleviates L-dopa–induced dyskinesia via lower D1R activity in Parkinson’s rats
Source: Aging (Albany NY). 2019 Dec 18;11(24):12315–27. doi: 10.18632/aging.102574 (PMC6949085; doi:10.18632/aging.102574)
Supplement: Supplementary Figures [file aging-11-102574-s001..pdf]

SUPPLEMENTARY FIGURES

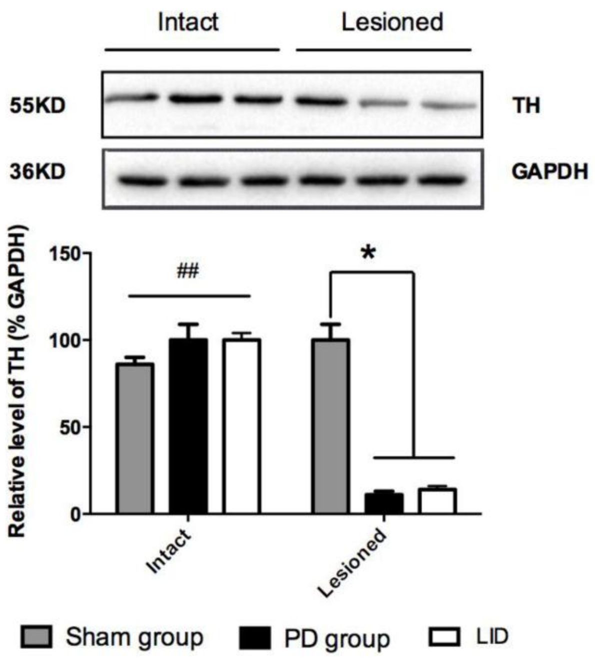

Supplementary Figure 1. Tyrosine hydroxylase (TH) protein levels in the Sham, PD and LID rats by western blot (n=4).

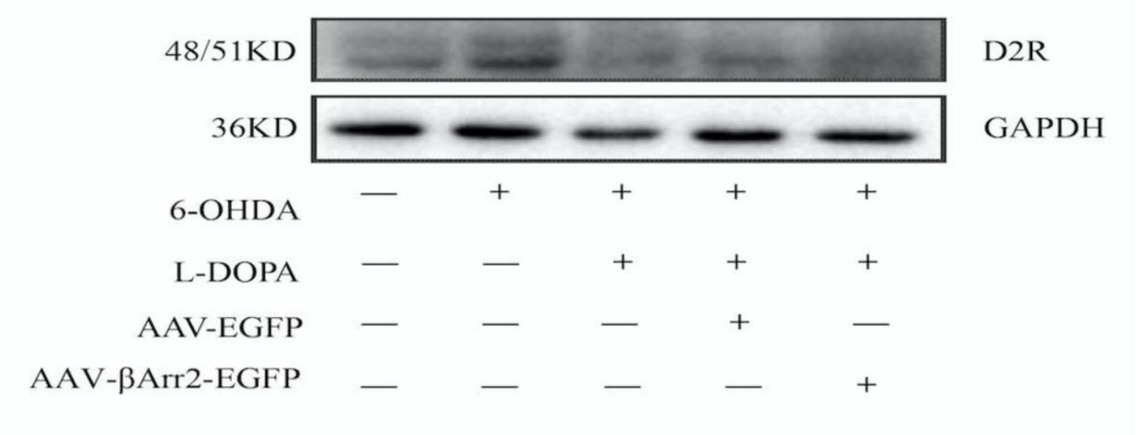

Supplementary Figure 2. D2R protein levels relative to GAPDH in each group by western blot (n=4).
